# Vitamin B_12_ is associated negatively with anemia in older Chinese adults with a low dietary diversity level: evidence from the Healthy Ageing and Biomarkers Cohort Study (HABCS)

**DOI:** 10.1186/s12877-023-04586-7

**Published:** 2024-01-04

**Authors:** Ling Liu, Jinhui Zhou, Chen Chen, Yingli Qu, Jun Wang, Feng Lu, Yingchun Liu, Jiayi Cai, Saisai Ji, Yawei Li, Heng Gu, Feng Zhao, Yuebin Lyu, Xiaoming Shi

**Affiliations:** 1 Hospital of Beijing Forestry University, Beijing, 100083 China; 2 China CDC Key Laboratory of Environment and Population Health, National Institute of Environmental Health, Chinese Center for Disease Control and Prevention, 7 Panjiayuan Nanli, Chaoyang, Beijing, 100021 China; 3 Beijing Municipal Health Commission Information Center, Beijing Municipal Health Commission Policy Research Center, Beijing, 100034 China

**Keywords:** Chinese older adults, Vitamin B_12_, Anemia, Dietary diversity score (DDS)

## Abstract

**Objective:**

The associations between plasma vitamin B_12_ level and anemia under different dietary patterns in elderly Chinese people are poorly understood. We aimed to examine the associations between plasma vitamin B_12_ levels and anemia under different dietary patterns in adults aged 65 years and older in nine longevity areas in China.

**Methods:**

A total of 2405 older adults completed a food frequency questionnaire at the same time as a face-to-face interview. The dietary diversity score (DDS) was assessed based on the food frequency questionnaire, with the low DDS group referring to participants with a DDS score ≤ 4 points. Vitamin B_12_ levels were divided into two groups of high (>295 pg/mL) and low (≤ 295 pg/mL) with the median used as the cut-off point. Sub-analyses were also performed on older adults divided into tertiles of vitamin B_12_ levels: low (< 277 pg/mL), medium (277–375 pg/mL) and high (> 375 pg/mL) to study the association of these levels with anemia.

**Results:**

Six hundred ninety-five (28.89%) of these people were diagnosed with anemia and had a mean age of 89.3 years. Higher vitamin B_12_ levels were associated with a decreased risk of anemia (multi-adjusted OR, 0.59, [95% CI, 0.45 ~ 0.77] *P* < 0.001) in older adults with a low DDS, whereas no significant association between vitamin B_12_ levels and anemia was found in older adults with a high DDS in a full-model after adjustment for various confounding factors (multi-adjusted OR, 0.88, [95% CI, 0.65 ~ 1.19], *P* = 0.41).

**Conclusion:**

The relationship between vitamin B_12_ levels and the prevalence of anemia was significant only when the level of dietary diversity in the older adults was relatively low. The dietary structure of the population should be taken into consideration in combination in order to effectively improve anemia status by supplementing vitamin B_12_.

## Key points

The relationship between vitamin B_12_ and the prevalence of anemia was significant only when the dietary structure of the population was relatively simple.

## Why does this paper matter?

The dietary structure of the population should be taken into consideration in combination in order to effectively improve anemia status by supplementing vitamin B_12_ which is more meaningful for public health practice.

## Introduction

Anemia is very prevalent in older adults, especially in those older than 85 years [1, 2]. As we know, vitamin B_12_ plays an important biological role in the hematopoietic process [3–5]. Megaloblastic anemia is a type of large cell anemia caused by several reasons including synthesis of deoxyribonucleic acid in bone marrow hematopoietic cells or the slow replication rate. However, vitamin B_12_ and folate deficiency were the most common cause of the disorder. An unhealthy diet, increased consumption of processed food products and digestive system dysfunction were the potential factors of vitamin B_12_ deficiency among the older adults. The diet of many older adults is always over emphasized because the prevalence of diabetes and hypertension in the aged is relatively high, or because they have a relatively simple dietary structure due to lifestyle and economic reasons, which may in the long term lead to vitamin B_12_ deficiency and nutritional megaloblastic anemia. Anemia and low plasma vitamin B_12_ concentrations are very common in older adults who are frequently administered vitamin B_12_ supplements. But some studies have reported that not all older people with anemia and subnormal vitamin B_12_ levels benefit from vitamin B_12_ supplementation alone and it has been suggested that trials are needed to verify whether this patient group should all be treated with hydroxocobalamin [6–9]. Anemia and vitamin B_12_ levels in older people are both closely related to dietary habits. According to the dietary guidelines in many countries, such as China, dietary diversity is one of the main characteristics of a healthy diet, with residents guided to implement “dietary diversification” and increase the variety of similar and different kinds of food in their diet. This approach ensures an adequate nutrient intake with dietary diversification improving the quality of the diet and leading to better nutritional and health status [10–12]. In practice, Dietary diversity score (DDS) is a good indicator and widely used for studying the relationship between dietary diversity and nutritional balance and health status. The majority of previous studies [6, 13, 14] have focused only on whether the occurrence of anemia is related to vitamin B_12_ deficiency, but have not paid attention to that if the different dietary patterns have an impact on their relationship. So there was still a controversy on the health benefits of vitamin B_12_ supplementation or only supplementing vitamin B_12_ without adjusting the structure of the diet, resulting in a limited improvement in anemia. Our research was conducted to investigate whether there were differences in the correlation between vitamin B_12_ deficiency and the prevalence of anemia under different dietary diversity levels.

## Materials and methods

### Study design and participants

**Fig. 1:**
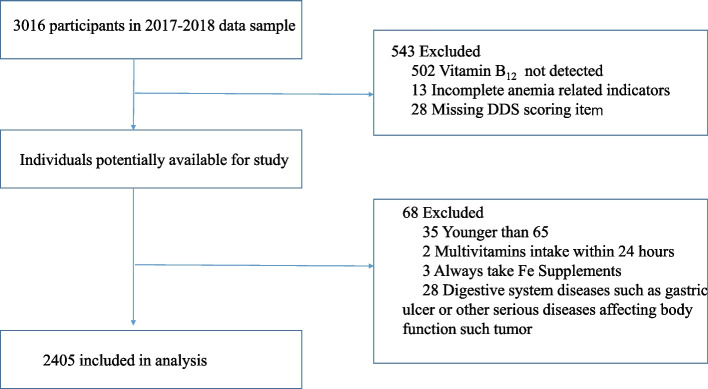
Flow chart of participants recruitment in study

Participants in the study had been enrolled in nine longevity areas from the Healthy Ageing and Biomarkers Cohort Study (HABCS) in China. we conducted a biomarker substudy of the 2017–2018 HABCS in nine longevity regions which covered the central, eastern and southern parts of China in 2017, The details of this survey have been described in a previous report [15]. As shown in Fig. 1, elderly people aged 65 or older who got vitamin B_12_ tested and had complete anemia related indicators and DDS scoring item were included. Elderly who took multivitamins within 24 hours or took Fe supplements regularly, or diagnosed with digestive system diseases such as gastric ulcer or other serious diseases affecting body function such tumor were excluded from the study. 2405 individuals aged 65 years or over included in the final data analysis. The study was approved by the biomedical ethics committee of National Institute of Environmental Health, Chinese Center for Disease Control and Prevention (IRB:201922). A signed informed consent form was obtained in writing from all subjects and/or their legal guardian(s).

All methods were carried out in accordance to relevant guideline and regulation.

A venous blood (4 mL volume) was collected from each subject. The hemoglobin (Hb) concentration of the subjects was measured by the cyaniding high iron method at a local laboratory. The remainder of the blood samples were sent to the clinical laboratory center of Capital Medical University for testing. The routine hematology tests included hemoglobin, blood cell count and mean corpuscular volume (MCV) that were measured with standard methods by a blood cytoanalyzer. Based on the World Health Organization (WHO) definition and a large study [16], we defined anemia as a Hb level < 130 g/L in men and < 120 g/L in women [17], and moderate/severe anemia as a Hb < 110 g/L in both genders. Morphological classification was performed according to the mean MCV and mean corpuscular hemoglobin contentration(MCHC). The values for the different hematological conditions were: megaloblastic anemia (MCV > 100 FL); normal cell anemia (MCV normal); simple microcytic anemia (MCHC ≥300 g/L and MCV < 80 FL); and hypochromic microcytic anemia (MCHC < 300 g/L and MCV < 80 FL) [18, 19]. As there is no established threshold for defining functional vitamin B_12_ deficiency [4, 20, 21] in older people, vitamin B_12_ status was divided into high (>295 pg/mL) and low (≤295 pg/mL) groups. Sub-analyses were also performed on older people with low (< 277 pg/mL), medium (277–375 pg/mL) and high (> 375 pg/mL) vitamin B_12_ levels to study the relationships between all these levels and anemia.

### Determination of vitamin B_12_ status

Plasma vitamin B_12_ concentrations were measured using chemiluminescent technology with a reference range of 150-738 pg/mL. Vitamin B_12_ levels were divided into two groups of high (>295 pg/mL) and low (≤ 295 pg/mL) by median. Sub-analyses were also performed on older adults divided into low (< 277 pg/mL), medium (277-375 pg/mL) and high (> 375 pg/mL) vitamin B_12_ level by tertiles.

### Assessment of DDS

A food frequency questionnaire survey was conducted at the same time as face-to-face interviews with all participants. DDS was evaluated according to a food frequency questionnaire [22, 23] that included nine major categories of food: meat, fish, beans, eggs, fruits, tea, nut, fresh vegetables and agaric. The consumption of these items was recorded as either “regularly or every day nearly”, “not regularly, but at least once/week”, “not every week, but at least once/month”, “not every month, but occasionally”, or “never or rarely”. As oil and cereals are consumed by almost all Chinese people they were not counted in the dietary diversity score [24]. The consumption of any food group as “regularly or every day nearly” or “not regularly, but at least once/week” intake was categorized as one DDS unit, while the other frequency intakes were not classified as a DDS unit. Because a total of nine categories of food were scored, 9 DDS points is the highest, representing the greatest diversity level. We set score ≥ 5 points as high DDS group and score ≤ 4 points as low DDS group. The DDS questionnaire used was developed to evaluate the balance of food intake and diet healthfulness and was composed of many kinds of healthy food items [25]. The scientific validity of the DDS questionnaire has been confirmed in previous studies [10, 24].

### Covariates

Some potential confounders were considered by a standardized and structured questionnaire including demographic variables such as residential region, nationality, age, gender, education status, marital status and health behaviors or habits information including current alcohol consumption levels, current smoking habits, self-reported measures of physical activity and physical health. After the face-to-face interview, all the individuals were required to attend a detailed health examinations including measurement of height and weight. All covariate information were defined as follows: demographic information including age (years), gender, residence (urban or rural),education background (illiteracy, primary school, junior middle school and above), body mass index (BMI, kg/m^2^) and current marital status (married or not); lifestyle behavior such as alcohol consumption (current drinker or not), smoking habits (current smoker or not), tea drinking habits (yes or no), exercise regularly (yes or no), use of multivitamin supplements (yes or no), prevalence of one or more of the following diseases including cerebrovascular, heart, digestive, respiratory and kidney disease (yes or no). Comorbidity was defined as the presence of more than one diseases listed above in a person in a defined period. The definition of high blood pressure for the aged was systolic blood pressure(SBP) was≥140 mmHg and/or diastolic blood pressure(DBP) was≥90 mmHg, or a self-reported diagnosed hypertension by a physician.

### Statistical analysis

We compared the continuous and categorical variables between different groups using Student’s T-test or the chi-square test respectively. Odds ratios (ORs) and 95% confidence intervals (CIs) were calculated for the relationship between vitamin B_12_ deficiency and anemia, with multiple logistic regression models used to adjust for possible confounders. Variables with a *P* ≤ 0.05 in the univariate analysis were regarded as likely predictors and mutually adjusted for in the multiple logistic regression models that included age, gender, level of education, nationality, residence, smoking habits, tea drinking habits, physic activity habits and BMI. To study the linear or curvilinear association between vitamin B_12_ and the odds of suffering from anemia, we performed generalized additive models. Sensitivity analyses were conducted by classifying vitamin B_12_ levels into three levels and modifying the outcomes of the study population to the diagnosis of severe anemia to evaluate the robustness of the results. All the statistical tests were performed with SAS 9.4 software (SAS Institute Inc., Cary, NC, USA), and were two-sided (*P* < 0.05).

## Results

1. Baseline characteristics of the older adults

The demographic description of the older adults grouped according to the type of anemia are summarized in Table 1. In total, 2405 older people were included in the study, 695 (28.80%) of whom were diagnosed with anemia with a mean age of 89.3 years. The average level of vitamin B_12_ among the aged with anemia was 308 pg/mL, which was lower than the level of 357 pg/mL in individuals without anemia (Table 1). The prevalence of anemia was 33.86% in older people with lower vitamin B_12_ levels (vitamin B_12_ < 295 pg/mL), which was 10% higher than that observed in those with a higher vitamin B_12_ level (vitamin B_12_ ≥ 295 pg/mL). However, in the older people with a low vitamin B_12_ level, the prevalence of anemia changed from 28.90% in the high DDS group to 38.14% in the low DDS group. In older people with a high vitamin B_12_ level, the prevalence of anemia changed to a lesser degree according to the DDS from 22.53% in the high DDS group to 25.31% in the low DDS group.

**Table 1:** Comparison of elderly characteristics based on anemia status

Factors	Non-anemia	Anemia	*t*|*χ2*	*P-*value
Age^a^(years)	82.5 ± 11	89.3 ± 10.6	4.38	< 0.001
Female^b^(%)	867 (50.7)	385 (55.4)	4.36	0.037
Nationality(han)^b^ (%)	1488 (93.2)	569 (87.1)	21.55	< 0.001
Married^b^(%)	870 (51.3)	214 (31)	82.06	< 0.001
Rural^b^(%)	1537 (94.5)	638 (96.2)	3.06	0.080
Education level^b^(%)				
Illiteracy	795 (47.6)	437 (65.5)	63.72	< 0.001
1-6 years	638 (38.2)	179 (26.8)		
≥7 years	239 (14.3)	51 (7.7)		
Smoking^b^(%)	327 (19.2)	90 (13)	13.14	< 0.001
Drinking^b^(%)	320 (18.8)	104 (15.1)	4.65	0.031
Physic^b^(%)	391 (23.2)	85 (12.5)	35.72	< 0.001
Tea drinking^b^(%)	1367 (81.1)	621 (90)	28.52	< 0.001
Disease^b^(%)	705 (41.2)	313 (45)	2.94	0.087
BMI^a^(kg/m^2^)	23 ± 4.4	21.4 ± 3.9	13.78	< 0.001
Elevated BMI^b^(%)	611 (37.1)	160 (24.7)	31.88	< 0.001
DDS^a^(point)	4.5 ± 1.9	4.10 ± 1.8	6.15	< 0.001
Low DDS^b^(%)	848 (50.2)	404 (58.5)	13.32	< 0.001
HGB^a^(g/L)	141.7 ± 13.2	110.7 ± 13.1	7.82	< 0.001
VB_12_^a^(pg/mL)	357.3 ± 181.8	307.9 ± 170.3	52.25	< 0.001

*BMI* Body mass index, *DDS* Dietary diversity score; *HGB* Hemoglobin

^a^ Continuous variables listed as mean ± SE including age,BMI,DDS,HGB, Vitamin B_12_,were compared between two groups using Student’s T test

^b^Dichotomous variables listed as n(%),including gender, race, marrage status,district,education level, smoking habits, tea drinking habits,physic activities,disease status,DDS group,BMI group,were compared between anemia and non-anemia groups using Chi-square tests

(2) The relationship between vitamin B_12_ status and anemia

Higher vitamin B_12_ levels can reduce the risk of anemia in the elderly (crude OR, 0.61 [95% CI, 0.51–0.73]; basic adjusted OR, 0.69 [95% CI, 0.57–0.83]; and multi-adjusted analysis that controlled for education level, race, smoking habits, tea drinking habits, and BMI, OR, 0.70[95% CI, 0.57–0.85]), We studied the association between vitamin B_12_ status and different types of anemia using logistic regression and found that the vitamin B_12_ status was only significantly associated with the prevalence of megaloblastic anemia and normal cell anemia (Tables 2 and 3).

**Table 2:** Logistic regression and subgroup analysis for association of anemia with VB12 status under different DDS

**Variables**	**Unadjusted**		**Basic model**^**a**^		**Final Model**^**b**^	
	**OR(*95%CI*)**	***P***	**OR(*95%CI*)**	***P***	**OR (*95%CI*)**	***P***
All (*N* = 2405)	0.61 (0.51–0.73)	<0.001	0.69 (0.57–0.83)	<0.001	0.70 (0.57–0.85)	<0.001
Low DDS group (*N* = 1252)	0.55 (0.43–0.70)	<0.001	0.59 (0.46–0.76)	<0.001	0.59 (0.45–0.77)	<0.001
DDS≤3(*N* = 799)	0.55 (0.41–0.75)	<0.001	0.6 (0.44–0.82)	0.002	0.62 (0.44–0.88)	0.007
DDS=4 (*N* = 453)	0.55 (0.36–0.83)	0.005	0.61 (0.40–0.94)	0.024	0.47 (0.29–0.77)	0.003
High DDS group (*N* = 1127)	0.72 (0.55–0.94)	0.015	0.85 (0.64–1.12)	0.245	0.88 (0.65–1.19)	0.411
DDS=5 (*N* = 475)	0.99 (0.67–1.40)	0.973	1.13 (0.74–1.72)	0.564	1.15 (0.73–1.83)	0.548
DDS≥6 (*N* = 652)	0.57 (0.39–0.81)	0.002	0.67 (0.45–0.99)	0.042	0.7 (0.45–1.08)	0.104
Gender						
The male low DDS group (*N* = 579)	0.58 (0.41–0.84)	0.003	0.64 (0.44–0.94)	0.021	0.62 (0.42–0.93)	0.027
The female low DDS group (*N* = 673)	0.52 (0.38–0.73)	<0.001	0.57 (0.40–0.80)	0.001	0.59 (0.41–0.85)	0.005
The male high DDS group (*N* = 559 )	0.74 (0.50–1.12)	0.152	0.90 (0.59–1.39)	0.644	0.98 (0.61–1.56)	0.945
The female High DDS group (*N* = 568 )	0.67 (0.47–0.96)	0.030	0.79 (0.54–1.15)	0.217	0.82 (0.55–1.23)	0.340
Low DDS group with age ≥ 80 (*N* = 830)	0.63 (0.48–0.84)	0.002	0.66 (0.50–0.89)	0.005	0.66 (0.48–0.90)	0.010
Low DDS group with age < 80 (*N* = 442)	0.42 (0.25–0.69)	0.001	0.43 (0.26–0.70)	0.001	0.43 (0.25–0.72)	0.002
High DDS group with age ≥ 80 (*N* = 652)	0.93 (0.67–1.28)	0.639	0.97 (0.7–1.35)	0.869	1.00 (0.70–1.42)	0.982
High DDS group with age < 80 (*N* = 475)	0.57 (0.33–0.98)	0.043	0.58 (0.33–1.00)	0.050	0.63 (1.13–2.93)	0.109

^a^Basic model,adjusted for age,gender

^b^Final model, adjusted for age, gender, race, education levels, current marital status, physic activity habits, smoking habits, tea drinking habits, BMI

**Table 3:** Logistic regression for association of different types of anemia with VB12 status under different DDS

**Variables**	**Unadjusted**		**Basic model**^**a**^		**Final Model**^**b**^	
	**OR(*95%CI*)**	***P***	**OR(*95%CI*)**	***P***	**OR(*95%CI*)**	***P***
All macrocytic anemia (*N* = 130)	0.55 (0.38–0.79)	0.001	0.62 (0.43–0.90)	0.011	0.66 (0.45–0.98)	0.038
Macrocytic anemia with low DDS (*N* = 77)	0.42 (0.25–0.71)	0.009	0.46 (0.27–0.77)	1.295	0.49 (0.29–0.84)	0.001
Macrocytic anemia with high DDS (*N* = 53)	0.75 (0.43–1.31)	0.312	0.89 (0.51–1.56)	0.680	0.98 (0.54–1.78)	0.941
All normocytic anemia (*N* = 483)	0.72 (0.59–0.88)	0.002	0.81 (0.66–1.00)	0.046	0.79 (0.63–0.98)	0.035
Normocytic anemia with low DDS (*N* = 275)	0.70 (0.53–0.92)	0.010	0.75 (0.57–1.00)	0.046	0.71 (0.53–0.96)	0.024
Normocytic anemia with high DDS (*N* = 208)	0.77 (0.57–1.04)	0.085	0.90 (0.66–1.23)	0.490	0.91 (0.66–1.27)	0.588
All microcytic anemia (*N* = 16)	1.02 (0.38–2.73)	0.969	1.12 (0.42–3.01)	0.826	1.16 (0.40–3.36)	0.782
Microcytic anemia with low DDS (*N* = 11)	0.99 (0.30–3.25)	0.983	1.08 (0.33–3.59)	0.896	1.06 (0.32–3.53)	0.921
Microcytic anemia with high DDS (*N* = 5)	0.78 (0.13–4.69)	0.786	0.76 (0.13–4.65)	0.768	0.62 (0.06–7.04)	0.703
All microcytic hypochromic anemia (*N* = 62)	0.48 (0.28–0.82)	0.007	0.52 (0.30–0.89)	0.018	0.59 (0.32–1.07)	0.083
Microcytic hypochromic anemia with low DDS (*N* = 41)	0.48 (0.24–0.95)	0.035	0.50 (0.25–1.00)	0.049	0.58 (0.27–1.24)	0.159
Microcytic hypochromic anemia with high DDS (*N* = 21)	0.52 (0.21–1.26)	0.148	0.60 (0.24–1.47)	0.263	0.60 (0.23–1.59)	0.306

^a^Basic model,adjusted for age,gender

^b^Final model, adjusted for age, gender, race, education levels, current marital status, physic activity habits, smoking habits, tea drinking habits, BMI

(3) The relationship between vitamin B_12_ status and anemia under low DDS

Vitamin B_12_ status was associated negatively with anemia in older adults with a low DDS (multi-adjusted analysis OR, 0.59 [95% CI, 0.45–0.77]). In contrast, the association between vitamin B_12_ levels and anemia in older people with a high DDS in the full-model after adjustment for various confounding factors was not statistically significant(multi-adjusted OR, 0.88, [95% CI, 0.65 ~ 1.19],*P* = 0.41).When we further subdivided the population groups according to the DDS score, the results were still consistent with the above. Subgroup analysis stratified were performed with the basic model, basic-adjusted model, or fully adjusted model. The relationship between vitamin B_12_ status and the prevalence of anemia including megaloblastic anemia and normal cell anemia was only significant in older people with a low DDS score (*P* < 0.001 and *P* < 0.024,respectively), The ORs were 0.49(0.29–0.84),0.71(0.53–0.96)respectively. The statistical results did not change with gender and age group (over 80 years old or under 80 years old) (Tables 2 and 3).

**Fig. 2:**
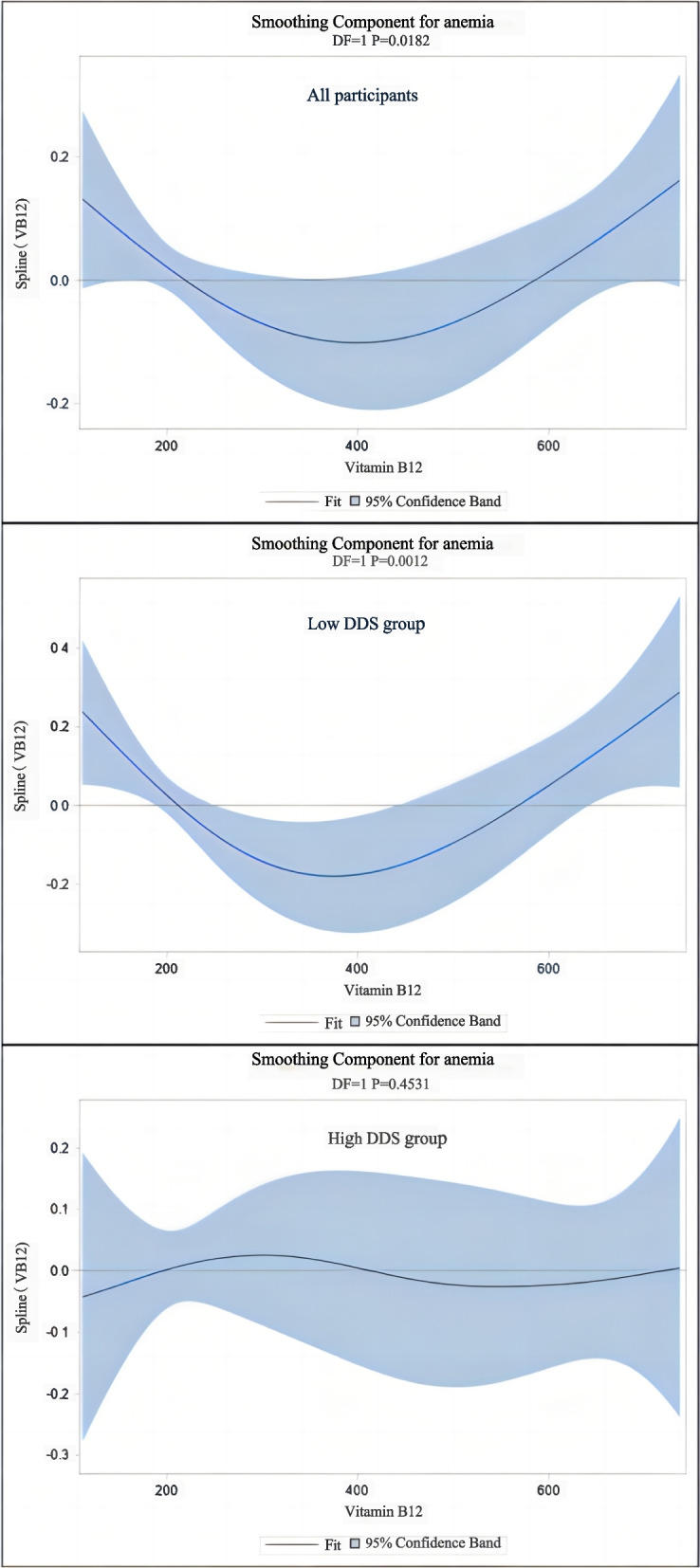
Smoothing component for anemia. *Notes:* Association of VB12 with anemia for the elderly, the Healthy Ageing and Biomarkers Cohort Study (HABCS). The lines estimated impact of VB12 on risk of anemia under different DDS among the elderly, and the shaded area indicates the 95% confidence intervals

As shown in Fig. 2, we also found a U-shaped association between anemia and vitamin B_12_ status in the GAMs models and the quadratic models in older adults with a low DDS score, but did not find a similar significant result in older adults with high DDS score. This indicates that older adults may have the lowest risk of overall anemia when their level of vitamin B_12_ is > 437.94 pg/mL. Among the participants whose vitamin B_12_ levels were simultaneously below the cut-point (438 pg/mL) and DDS score below 5 points, there was a trend towards a significant decrease in the risk of anemia with increasing levels of vitamin B_12_. Each 1 pg/mL increase in vitamin B_12_ level corresponded to a 0.4% lower risk of anemia after adjusting for covariates in the full-model (OR, 0.996 [95%CI, 0.994–0.998]). Compared with participants with lower vitamin B_12_ levels (≤238 pg/ mL) those with a higher vitamin B_12_ level (> 238 pg/ mL) had a 36% lower risk of anemia in the full-model (OR, 0.64[95%CI, 0.47–0.86]). However, such a negative or positive association was not observed in participants whose vitamin B_12_ levels were above 438 pg/ mL or had a DDS score ≥ 5 points, regardless of whether vitamin B_12_ was considered as either a continuous or categorical variable.

(4) Results of the sensitivity analyses

The results of the sensitivity analyses suggested that our findings were relatively robust and did not show material changes after categorizing the vitamin B_12_ levels into three levels and modifying the outcome of the study population according to the diagnosis of severe anemia in order to study the relationship between vitamin B_12_ status and anemia using logistic regression (Table 4).

**Table 4:** Sensitive analysis for the association of anemia with vitamin B_12_ status

**Variables**	**Unadjusted**		**Basic model**^**a**^		**Final Model**^**b**^	
	**OR(*95%CI*)**	***P***	**OR(*95%CI*)**	***P***	**OR(*95%CI*)**	***P***
All anemia (*N*=291)						
Low B12 (<277pg/mL)	1.0 (ref.)		1.0 (ref.)		1.0 (ref.)	
Medium B12 2 (277–375pg/mL)	0.69 (0.54–0.87)	0.002	0.73 (0.57–0.93)	0.011	0.76 (0.58–0.99)	0.039
High B12 (>375pg/mL)	0.53 (0.43–0.65)	<0.001	0.62 (0.5–0.76)	<0.001	0.63 (0.50–0.79)	<0.001
Low DDS group (*N*=404 )						
Low B12 (< 277pg/mL)	1.0 (ref.)		1.0 (ref.)		1.0 (ref.)	
Medium B12 (277–375pg/mL)	0.54 (0.39–0.74)	<0.001	0.55 (0.–0.77)	0.001	0.56 (0.39–0.79)	0.001
High B12 (>375pg/mL)	0.48 (0.36–0.64)	<0.001	0.53 (0.39–0.71)	<0.001	0.53 (0.39–0.72)	<0.001
DDS ≤ 3 (*N*=271)						
Low B12 (< 277pg/mL)	1.0 (ref.)		1.0 (ref.)		1.0 (ref.)	
Medium B12 (277–375pg/mL)	0.57 (0.39–0.84)	0.0048	0.6 (0.40–0.90)	0.014	0.62 (0.39–0.97)	0.038
High B12 (>375pg/mL)	0.47 (0.33–0.67)	<0.0001	0.53 (0.36–0.77)	0.001	0.55 (0.36–0.84)	0.005
DDS=4 (*N*=133)						
Low B12 (< 277pg/mL)	1.0 (ref.)		1.0 (ref.)		1.0 (ref.)	
Medium B12 (277–375pg/mL)	0.5 (0.28–0.88)	0.0157	0.52 (0.29–0.94)	0.030	0.45 (0.23–0.88)	0.019
High B12 (>375pg/mL)	0.52 (0.32–0.83)	0.0065	0.57 (0.35–0.94)	0.026	0.42 (0.24–0.74)	0.003
High DDS group (*N*=287)						
Low B12 (< 277pg/mL)	1.0 (ref.)		1.0 (ref.)		1.0 (ref.)	
Medium B12 (277–375pg/mL)	0.95 (0.66–1.35)	0.766	1.04 (0.72–1.51)	0.837	1.16 (0.78–1.73)	0.463
High B12 (>375pg/mL)	0.62 (0.46–0.85)	0.003	0.78 (0.56–1.07)	0.123	0.80 (0.57–1.14)	0.215
DDS=5 (*N*=134)						
Low B12 (< 277pg/mL)	1.0 (ref.)		1.0 (ref.)		1.0 (ref.)	
Medium B12 (277–375pg/mL)	1.20 (0.71–2.04)	0.4934	1.38 (0.79–2.4)	0.263	1.32 (0.71–2.43)	0.381
High B12 (>375pg/mL)	0.97 (0.61–1.53)	0.8909	1.13 (0.70–1.83)	0.616	1.11 (0.65–1.88)	0.710
DDS≥6 (*N*=153)						
Low B12 (< 277pg/mL)	1.0 (ref.)		1.0 (ref.)		1.0 (ref.)	
Medium B12 (277–375pg/mL)	0.77 (0.48–1.25)	0.2956	0.83 (0.50–1.37)	0.461	1.10 (0.63–1.94)	0.733
High B12 (>375pg/mL)	0.46 (0.30–0.69)	0.0002	0.57 (0.37–0.89)	0.014	0.61 (0.37–1.00)	0.050
All severe anemia (*N*=250)						
Low B12 (< 277pg/mL)	1.0 (ref.)		1.0 (ref.)		1.0 (ref.)	
Medium B12 (277–375pg/mL)	0.55 (0.38–0.82)	0.003	0.57 (0.39–0.86)	0.007	0.63 (0.41–0.96)	0.033
High B12 (>375pg/mL)	0.68 (0.51–0.91)	0.011	0.80 (0.59–1.09)	0.158	0.81 (0.58–1.14)	0.226
Severe anemia with low DDS group (*N*=157)						
Low B12 (< 277pg/mL)	1.0 (ref.)		1.0 (ref.)		1.0 (ref.)	
Medium B12 (277–375pg/mL)	0.43 (0.26–0.72)	0.001	0.44 (0.26–0.74)	0.002	0.47 (0.27–0.83)	0.009
High B12 (>375pg/mL)	0.63 (0.43–0.94)	0.024	0.71 (0.47–1.06)	0.092	0.70 (0.46–1.08)	0.110
Severe anemia with high DDS group (*N*=93)						
Low B12 (< 277pg/mL)	1.0 (ref.)		1.0 (ref.)		1.0 (ref.)	
Medium B12 (277–375pg/mL)	0.94 (0.52–1.70)	0.830	1.05 (0.57–1.94)	0.880	1.21 (0.82–2.33)	0.560
High B12 (>375pg/mL)	1.03 (0.64–1.64)	0.919	1.36 (0.82–2.23)	0.231	1.39 (0.82–2.37)	0.224

^a^Basic model, adjusted for age, gender

^b^Final model, adjusted for age, gender, race, education levels, current marital status, physic activity habits, smoking habits, tea drinking habits, BMI

A significant association between vitamin B_12_ levels and severe anemia was only observed in older people with a low DDS (OR,0.47(0.27–0.83), *P* < 0.009.

## Discussion

We observed a negative relationship between vitamin B_12_ status and anemia which only remained significant in older people with a low DDS but not in those with a high DDS. We also found a U-shaped association between anemia and vitamin B_12_ status in the GAMs models and quadratic models in older people with a low DDS score, but did not find a similar significant result in older people with high DDS score. When the vitamin B_12_ level was close to 438 pg/mL the risk of anemia was lowest when analyzed by smoothing function fitting.

The negative association between vitamin B_12_ and anemia was only found among the participants who simultaneously had a vitamin B_12_ level below the cut-point(438 pg/mL) and a DDS score below 5 points at the same time. We therefore speculate that when the elderly have high levels of vitamin B_12_ or diversified diets, the risk of anemia don’t always further decrease with an increase of vitamin B_12_ level. Zhou [26] considered that iron deficiency anemia due to a lack of iron makes vitamin B_12_ unable to participate in the synthesis of erythrocyte DNA, causing iron deficiency anemia and increasing the serum levels of vitamin B_12_. This may obscure the real relationship between vitamin B_12_ levels and anemia especially when the serum vitamin B_12_ level is high. Because of the nondeterminacy of the dose relationship between vitamin B_12_ depletion and the onset of various diseases and the various laboratory methods for measuring vitamin B_12_, functional vitamin B_12_ deficiency is difficult to define. No universal gold standard was set for diagnosing cobalamin deficiency and the criteria for dividing older people into deficient or sufficient groups remains unclear [7, 27]. However, the following guidelines were used by some clinicians to classify cobalamin levels: < 200 pg/mL, deficiency is present, 200-300 pg/mL, deficiency possible, and > 300 pg/mL, deficiency is unlikely [28]. Our study classified older people into subnormal and normal groups according to the median or percentile of vitamin B_12_ status. The median value of vitamin B_12_ used in our study was 295 pg/mL, which is similar to the guidelines mentioned above. In many other recent studies in Western societies the proportion of people with a high vitamin B_12_ level was greater than that of people with a subnormal vitamin B_12_ level, with a median level of 392 pg/mL or higher [29–32]. However, the results of studies in elderly Chinese people showed that the level of vitamin B_12_ was relatively low and that the levels varied greatly in different studies [33, 34]. A study in people living in Southern China [35] reported that the adjusted mean level of plasma vitamin B_12_ was 260 pmol/L (352 pg/mL), while the level of people living in Northern China was 189 pmol/L (256 pg/mL), with levels tending to decrease with age. Our study showed that the mean age of older people with a low plasma vitamin B_12_ level was greater than that of those with a high level, indicating that older people are more likely to have vitamin B_12_ deficiency.

Serum or plasma vitamin B_12_ deficiencies are listed as established causes of anemia [36, 37], with many previous studies having investigated the relationship between vitamin B_12_ status and anemia. Most of these researches have focused on the negative association between vitamin B_12_ status and higher risk of macrocytic anemia, with only a few studies exploring the relationship between vitamin B_12_ status and different types of anemia. In addition, few studies have studied whether the lower vitamin B_12_ levels can increase the risk of different types of anemia by including dietary structure in the analyses.

The progress of DNA synthesis, erythropoiesis, and cell division, vitamin B_12_ is an important essential micronutrient. Therefore, DNA replication, cell division and S-phase progression in erythroblasts can be influenced by vitamin B_12_ deficiency [38]. Patients with very low levels of vitamin B_12_ may suffer from macrocytosis and anemia because of overproduction of protein in erythroblasts [39]. Vitamin B_12_ cannot be synthesized in the body and is completely dependent on dietary sources, especially all kinds of meat [40]. A systematic review of 25 studies on vitamin B_12_ deficiency and anemia in the aged showed inconsistent results [7]. There were three possible explanations why we observed a negative relationship between vitamin B_12_ status and anemia which was only remained significant in older people with a low DDS not in that with a high DDS. First, an inadequate dietary intake of foods rich in vitamin B_12_, such as animal products and food-cobalamin malabsorption (FCM) were the main causes of vitamin B_12_ deficiency [34]. Some studies have reported that malabsorption primarily affects people aged 60 years and older. About 40% of patients with an unexplained low serum vitamin B_12_ level may have FCM [41]. Although the causes for FCM are unclear, FCM explains why vitamin B_12_ depletion occurs along with aging and was the most common cause of vitamin B_12_ deficiency in the elderly population. The most probable factors that contribute to malabsorption of vitamin B_12_ in older people include intestinal microbial proliferation, chronic alcoholism, gastric reconstruction, pancreatic enzyme deficiency, sjogren syndrome and long-term ingestion of drugs such as antacids, biguanides, H_2_ receptor antagonists and proton pump inhibitors [42, 43]. If the older people had a balanced diet, but the vitamin B_12_ status remained low, it suggests that they may not only have vitamin B_12_ deficiency, but often accompanied by intestinal absorption and intestinal immunity problems. Then, malabsorption could lead to other anemia related deficiencies caused by Fe or folic acid, which are also important causes of nutritional anemia [44, 45]. This may disturb the relationship between vitamin B_12_ level and anemia. We know that because iron deficiency causes microcytosis and vitamin B_12_ deficiency causes macrocytosis, if patients suffer from concomitant deficiencies in iron and vitamin B_12_ at the same time, they may present with anemia with normal-sized RBCs (normocytic anemia) instead [46, 47]. The second explanation is that anemia due to nutritional deficiency is an acquired problem because of the insufficient quantity of bioavailable essential hematopoietic nutrients such as Fe, vitamin B_12_ and folic for red blood cell and hemoglobin synthesis. The older people with a high DDS had a balanced dietary intakes of various nutrients, which may promote their absorption to a certain extent.

Because the synthesis of red blood cells also involves the effective utilization of various nutrients, even if the level of vitamin B_12_ status is relatively low, a balanced diet may improve the bioavailability of vitamin B_12_ level in the process of erythropoiesis, so as to reduce the actual risk of anemia and modify the relationship between vitamin B_12_ level and anemia [48]. The third explanation is that in addition to the insufficient intake of vitamin B_12_, the older adults with a low DDS score also have insufficient intake of other anemia related nutrients. For example, vegetarians may have insufficient Fe intake, which further increases the possibility of anemia [49]. The dietary variety and the absorption capacity of various nutrients always declines with advancing age [50]. Dietary nutritional habits, vitamin B_12_, and serum iron deficiency therefore influence each other and in combination also affect the type of anemia that may develop. Low vitamin B_12_ and symptoms due to vitamin B_12_ deficiency especially neurological which can be irrevesible are usually corrected with supplements [51]. However, the effect of this supplementation on haematological parameters in community-dwelling older people with low vitamin B_12_ is not consistent [9], not all vitamin B_12_ administration can effectively improve the haemoglobin concentrations and MCV among older people [8, 9, 52]. Some other causes such as chronic inflammation, other hematological diseases, polypharmacy may play a role in the development of vitamin B_12_ deficiency and anaemia [53]. Sometimes multiple co-existing deficiencies of other nutrients such as Fe and folic acid may interfere with the clinical symptoms of vitamin B_12_ deficiency. Currently, there is no an independent gold standard for unequivocal characterization of vitamin B_12_ deficiency nor an agreement on how to treat (in relation to cobalamin preparation, dosage, formulation, intensity and duration of treatment), or on how and when to monitor the effects of vitamin B_12_ supplementation [43, 54]. Therefore, we should not only directly and blindly supplement vitamin B_12_ to improve the anemia symptoms of vitamin B_12_ deficient among older people. Both the causes of vitamin B_12_ deficiency and the dietary structure of the population need to be taken into consideration to decide whether to supplement vitamin B_12_ alone or associated with nutritional intervention for FCM prevention and treatment is needed.

### Strengths

To date, most previous studies on whether the anemia was associated with vitamin B_12_ deficiency were carried out in Western populations whose dietary habits are very different from those in China. The vitamin B_12_ level and prevalence of anemia in the elderly Chinese population are also different from that of other countries. It is therefore very important to carry out further analyses on the dietary habits of older people in China. The large number of people selected for our study lived in nine provinces in Northern and Southern China and were representative of the population in these areas. By combining dietary diversity with the relationship between vitamin B_12_ levels and different types of anemia in our analyses and a negative association between vitamin B_12_ and anemia was only found among the participants whose vitamin B_12_ levels were below the cut-point(438 pg/ mL) and DDS score were below 5 points at the same time, we consider that the results may be more meaningful for public health practice.

### Limitations

Our study has several limitations. Firstly, it’s a cross-sectional design .Furthermore, the fact that classification of the dietary score can be further refined. Thirdly, our evaluation of the vitamin B_12_ levels was based only on plasma vitamin B_12_ concentration. Concomitant deficiencies in cobalamin and folate are often found although we did not measure vitamin B_12_ metabolites and other anemia-related dietary nutrients including folic acid and serum iron which are closely related to dietary nutrition and anemia.

## Conclusions

We found that the relationship between vitamin B_12_ and the prevalence of anemia, mainly macrocytic anemia and normal cell anemia was only statistically significant when the dietary structure of the elderly population was relatively simple. In addition, a negative association between vitamin B_12_ and anemia was only observed among the participants who simultaneously had a vitamin B_12_ level below the cut-point and a DDS score below 5 points. In contrast, if the level of dietary diversity in the elderly was relatively high. The dietary structure of the population, the vitamin B_12_ levels and the type of anemia should therefore be taken into consideration together in order to improve anemia status by supplementing vitamin B_12_. We encourage further study on these relationships and hope our findings will both inform continuing debate on vitamin B_12_ and anemia under different dietary diversity levels and influence efforts to treat anemia in older people from a perspective of low vitamin B_12_ status under different dietary diversity levels.

## Data Availability

This study was based on the datasets from the Healthy Ageing and Biomarkers Cohort Study(HABCS). The datasets generated and analysed during the current study are not publicly available due the data shared by multiple researchers but are available from the corresponding author on reasonable request.
